# Akute virale Bronchiolitis und obstruktive Bronchitis bei Kindern

**DOI:** 10.1007/s00112-020-00993-x

**Published:** 2020-08-17

**Authors:** Christina Schorlemer, Ernst Eber

**Affiliations:** Klinische Abteilung für Pädiatrische Pulmonologie und Allergologie, Univ.-Klinik für Kinder- und Jugendheilkunde, Medizinische Universität Graz, Auenbruggerplatz 34/2, 8036 Graz, Österreich

**Keywords:** Respiratorisches Synzytialvirus, Risikofaktoren, Sauerstoff, β_2_-Agonisten, Asthma, Respiratory syncytial virus, Risk factors, Oxygen, Beta‑2 agonists, Asthma

## Abstract

Akute Bronchiolitis und obstruktive Bronchitis sind im Säuglings- und Kleinkindalter sehr häufige Krankheitsbilder. Sie werden durch Viren, v. a. respiratorisches Synzytialvirus und Rhinoviren, verursacht. Risikofaktoren für schwere Verläufe sind u. a. Frühgeburtlichkeit, Tabakrauchexposition und Immundysfunktionen. Die Diagnose kann durch Anamnese und klinische Untersuchung gestellt werden; Thorax-Röntgen und Laboruntersuchungen sind in der Regel nicht notwendig. Für die akute Bronchiolitis wurden viele therapeutische Ansätze propagiert, generell empfohlen sind aber nur supportive Maßnahmen (minimales Handling, Sicherstellung ausreichender Oxygenierung und Hydratation). Routinemäßig nicht empfohlen werden u. a. Antibiotika, Bronchodilatatoren, Kortikosteroide und Leukotrienrezeptorantagonisten. Kurz wirksame β_2_-Agonisten sind Therapie der 1. Wahl bei akuter obstruktiver Bronchitis. Bei häufigen und/oder schweren obstruktiven Bronchitiden kann zur Symptomkontrolle eine Therapie mit inhalativen Kortikosteroiden versucht werden. Die Entstehung von Asthma bronchiale ist jedoch durch keine medikamentöse Therapie zu verhindern.

## Abstract (en)

Acute viral bronchiolitis and wheezy bronchitis are very common disorders in infants and preschool children. They are caused by viruses, particularly by respiratory syncytial virus and rhinoviruses. Risk factors for severe disease include premature birth, tobacco smoke exposure and immune dysfunctions. Patient history and a physical examination are sufficient to establish a diagnosis; chest X-ray and laboratory investigations are not routinely necessary. For acute bronchiolitis, many treatment options have been suggested but only supportive measures, such as minimal handling, securing adequate oxygenation and hydration are generally recommended. Antibiotics, bronchodilators, corticosteroids und leukotriene receptor antagonists are not routinely recommended. Short-acting beta-2 agonists are the first choice for the treatment of wheezy bronchitis. Inhaled corticosteroids may be tried to control symptoms in children with frequently recurring and/or severe episodes of wheezy bronchitis. The development of bronchial asthma, however, cannot be prevented by any pharmacologic tretament.

## Lernziele

Nach der Lektüre dieses Beitrags

- kennen Sie die Unterschiede zwischen den und die Gemeinsamkeiten der beiden Krankheitsbilder akute Bronchiolitis und akute obstruktive Bronchitis.
- wissen Sie, welche Faktoren mit einem erhöhten Risiko für schwere Krankheitsverläufe assoziiert sind.
- kennen Sie wichtige diagnostische Schritte und Differenzialdiagnosen.
- können Sie zwischen sinnvollen und unnötigen bzw. ineffizienten therapeutischen Maßnahmen differenzieren.

## Hintergrund

Die akute virale Bronchiolitis und obstruktive Bronchitis sind sehr häufige Krankheitsbilder. Im 1. Lebensjahr sind akute Bronchiolitiden der häufigste Grund für Hospitalisierungen, und jedes 3. Kind hat zumindest einmal eine obstruktive Bronchitis vor dem vollendeten 3. Lebensjahr. Trotz der Häufigkeit ihres Auftretens existieren in der Praxis manche Unklarheit und auch Uneinigkeit, beginnend bereits mit der Definition dieser Erkrankungen und darüber hinaus bezüglich sinnvoller diagnostischer Schritte und effektiver therapeutischer Maßnahmen.

## Bronchiolitis

### Definition

Eine international einheitliche Definition der akuten viralen Bronchiolitis existiert nicht [1]. In den meisten Ländern Europas sowie in Australien und Neuseeland wird eine **virale Infektion**

virale Infektion

der unteren Atemwege im Säuglingsalter als akute Bronchiolitis bezeichnet; der Altersgipfel liegt in den ersten 6 Lebensmonaten [2]. In dieser Definition wird der Begriff akute Bronchiolitis manchmal auch nur für die erste derartige Erkrankung verwendet [1]. Der charakteristische Auskultationsbefund ist ein vorwiegend **endinspiratorisches Knisterrasseln**

endinspiratorisches Knisterrasseln

(„crackles“), verursacht durch den erhöhten Strömungswiderstand in den mit Sekret gefüllten distalen Bronchiolen. Ein pfeifendes bzw. giemendes exspiratorisches Geräusch („wheezing“) ist nur in manchen Fällen vorhanden [2]. In vielen Leitlinien werden als klinische Charakteristika der akuten Bronchiolitis eine vorangegangene bzw. bestehende Infektion der oberen Atemwege, Husten, Zeichen einer vermehrten Atemarbeit und verminderte Nahrungsaufnahme angeführt [1]. Sehr junge Säuglinge (v. a. Frühgeborene) können sich auch ausschließlich mit **zentralen Apnoen**

zentralen Apnoen

präsentieren [3, 4]. In Nordamerika wird der Begriff akute Bronchiolitis für jede virale Infektion der unteren Atemwege mit Obstruktion (auch bei nur vorhandenem „wheezing“) in den ersten 2 Lebensjahren verwendet. Dadurch kommt es zu einer Überschneidung mit dem im deutschsprachigen Raum als obstruktive Bronchitis klassifizierten Krankheitsbild. Durch die Heterogenität der Definition einer akuten Bronchiolitis werden die Interpretation und der Vergleich von klinischen Studien erschwert [2].

### Ätiologie und Epidemiologie

Im Säuglingsalter ist die akute Bronchiolitis die häufigste virale Infektion der unteren Atemwege [2]. Je nach Definition wird die Prävalenz von akuten Bronchiolitiden im 1. Lebensjahr mit 18–32 % beschrieben [5]. Die höchste Inzidenz besteht zwischen dem 3. und 6. Lebensmonat [3]. In Nordamerika werden 2–3 % aller Kinder im 1. Lebensjahr mit der Diagnose akute Bronchiolitis stationär aufgenommen [6]. Somit ist die akute Bronchiolitis der häufigste Grund für eine Hospitalisierung in dieser Altersgruppe [7]. Die Mortalität ist jedoch in ressourcenreichen Ländern wie Nordamerika äußerst gering [3]. Das humane **respiratorische Synzytialvirus**

respiratorische Synzytialvirus

(RSV) ist die Hauptursache von akuten Bronchiolitiden und kann bei 50–80 % der hospitalisierten Patienten isoliert werden [6]. Als zweithäufigster Erreger wird **humanes Rhinovirus**

humanes Rhinovirus

im nasopharyngealen Sekret nachgewiesen. Seltener detektiert werden Parainfluenzavirus, humanes Metapneumovirus, Coronavirus, Adenovirus, Influenzavirus oder Enterovirus [6]. Je nach Studie konnten bei 6–30 % von hospitalisierten Kindern mit akuter Bronchiolitis gleichzeitig mehrere Viren in den Atemwegen nachgewiesen werden. Nicht eindeutig ist die Datenlage, ob es bei viraler Koinfektion (zumeist RSV und Rhinovirus [5]) zu schwereren Krankheitsverläufen kommt [6, 7].Des Weiteren wurde gezeigt, dass bei bis zu 30 % der symptomfreien Kinder im Alter unter 6 Jahren zumindest ein respiratorisches Virus nachweisbar ist, sodass ein Virusnachweis auch einer prolongierten Virusausscheidung nach Infektion oder einer asymptomatischen Kolonisation entsprechen könnte [4, 7]. Der Nachweis von RSV korreliert fast immer mit einer akuten Erkrankung [4].

In den meisten Teilen Europas (entsprechend der gemäßigten nördlichen Hemisphäre) treten durch RSV verursachte akute Bronchiolitiden vorwiegend in den **Wintermonaten**

Wintermonaten

auf, typischerweise von Ende Oktober bis April mit einem Gipfel der Erkrankungsfälle im Januar und im Februar [7]. Möglicherweise haben auch wetterbedingte Faktoren (wie das Einatmen trockener, kalter Luft und das vermehrte Aufhalten in Innenräumen) einen Einfluss auf die Virusausbreitung und Schwere der Erkrankung [6, 7]. Durch Rhinoviren verursachte Erkrankungsfälle werden v. a. im Herbst und Frühling beobachtet und sind mit kürzeren Krankenhausaufenthalten assoziiert als RSV-induzierte akute Bronchiolitiden [6].

### Pathogenese und Pathophysiologie

Neben direkt zytopathischen Effekten der krankheitsverursachenden Viren spielen in der Pathogenese der akuten Bronchiolitis v. a. lokale und systemische (zelluläre) Immunreaktionen eine Rolle. Die Variabilität der **individuellen Immunreaktionen**

individuellen Immunreaktionen

stellt die Basis für unterschiedlich schwere Krankheitsverläufe dar. Es wird vermutet, dass eine neurogen vermittelte Immunantwort für eine erhöhte bronchiale Reagibilität nach einer RSV-Infektion verantwortlich ist [2].

Die akute Bronchiolitis ist gekennzeichnet durch **Nekrose**

Nekrose

respiratorischer Epithelzellen, gefolgt von einer ausgeprägten entzündlichen Reaktion mit Ödem der Atemwege und des umgebenden Gewebes sowie einer verstärkten Sekretproduktion. Abgeschilferte nekrotische Epithelzellen und Sekret führen zu einer partiellen oder totalen **Obstruktion**

Obstruktion

der Bronchiolen; daraus resultieren Lungenabschnitte mit Überblähung (durch den Ventilmechanismus bei partieller Atemwegsobstruktion, der auch das typische endinspiratorische Knisterrasseln erklärt) oder Atelektasen (durch Wegfall der Belüftung bei totaler Atemwegsobstruktion). Beide Phänomene resultieren in einem Ventilation-Perfusion-Missverhältnis und führen konsekutiv zu **Hypoxämie**

Hypoxämie.

Bei schweren Verläufen kann die durch Atemwegsobstruktion erhöhte Atemarbeit zu **respiratorischer Erschöpfung**

respiratorischer Erschöpfung

führen. Zentrale Apnoen können als Resultat einer direkten Affektion des Atemzentrums durch eine RSV-Infektion auftreten [2, 7].

### Symptome und klinischer Verlauf

Zunächst tritt ein **Prodromalstadium**

Prodromalstadium

mit Zeichen und Symptomen einer viralen Infektion der oberen Atemwege (verlegte Nasenatmung, Rhinorrhoe und in etwa 30 % der Fälle Fieber unter 39 °C) auf [7]. Es folgt nach 24–72 h eine Infektion der unteren Atemwege [3]. Persistierender Husten, Tachypnoe und Zeichen einer vermehrten Atemarbeit (interkostale, subkostale oder supraklavikuläre Einziehungen, Nasenflügeln, Einsatz der Atemhilfsmuskulatur) sind typische Zeichen und Symptome [7]. Der charakteristische Auskultationsbefund ist inspiratorisches Knisterrasseln, während „wheezing“ nur in manchen Fällen vorhanden ist [2]. Je nach Schweregrad der akuten Bronchiolitis kann es typischerweise nach 3 bis 5 Krankheitstagen zu einer Einschränkung der Ernährung bis hin zur Trinkunfähigkeit und **Dehydratation**

Dehydratation

kommen [2, 3]. Bei schweren Verlaufsformen kann eine respiratorische Insuffizienz auftreten, sodass intensivmedizinische Maßnahmen und ggf. ein respiratorischer Support notwendig werden; diese Fälle treten aber sehr selten auf [2, 4]. Bei Säuglingen in den ersten 2 Lebensmonaten, insbesondere bei Frühgeborenen, kann sich eine akute Bronchiolitis einzig durch Apnoen manifestieren [6]. Typisch für das klinische Erscheinungsbild von akuten Bronchiolitiden ist, dass die Symptomatik besonders am Krankheitsbeginn von Minute zu Minute sehr variabel ist, wodurch eine Einschätzung des Schweregrads der Erkrankung schwierig sein kann [7]. Im Schnitt sistieren die Symptome nach 14 Tagen, 10 % der Kinder mit akuter Bronchiolitis weisen nach 3 Wochen noch Husten auf [3, 7].

### Diagnose

Die Diagnose akute Bronchiolitis wird auf Basis der Anamnese und klinischen Untersuchung gestellt; auch der Schweregrad der Erkrankung wird so ermittelt [4, 7]. Da die klinischen Befunde oft binnen Minuten sehr variieren, sollten sie mehrfach über einen Beobachtungszeitraum erhoben werden [7]. Nach Absaugen der Nase kann die Symptomatik vonseiten der unteren Atemwege möglicherweise besser beurteilt werden [7]. Wenn möglich, soll bei allen Kindern mit Verdacht auf akute Bronchiolitis eine Messung der Sauerstoffsättigung mithilfe der **Pulsoxymetrie**

Pulsoxymetrie

erfolgen [3]. Zur **Atemfrequenzbestimmung**

Atemfrequenzbestimmung

ist das Zählen der Atemzüge über die Dauer einer Minute genauer als bei kürzeren Intervallen [4]. Die Bestimmung von Atemfrequenz und Sauerstoffsättigung bei Raumluft sowie die Beurteilung von **thorakalen Einziehungen**

thorakalen Einziehungen

und der Ernährungssituation erlauben eine Einteilung der akuten Bronchiolitis in 3 Schweregrade (Tab. 1; [2]).

**Table 1:** 

Befund	Leicht	Mittel	Schwer
Atemfrequenz/min	<40	40–70	>70
O_2_-Sättigung unter Raumluft (%)	>92	88–92	<88
Sternale/thorakale Einziehungen	Fehlend	+	++
Ernährung	Problemlos	Schwierig	Unmöglich

#### Cave

Besonders bei Krankheitsbeginn können die klinischen Befunde von Minute zu Minute sehr variabel sein. 

#### Bildgebende Diagnostik

Da der Röntgenbefund schlecht mit dem Schweregrad und dem Erkrankungsverlauf korreliert, wird ein **Thoraxröntgen**

Thoraxröntgen

nicht in allen Fällen empfohlen [6]. Während das radiologische Bild einer Pneumonie ähneln kann, ist die Prävalenz einer gesicherten Pneumonie im Rahmen einer akuten Bronchiolitis niedrig [3, 7]. Die Unterscheidung zwischen Atelektasen und Infiltraten kann schwierig sein [2]. Es konnte wiederholt gezeigt werden, dass nach routinemäßiger Durchführung von Thoraxröntgen mehr Antibiotika verordnet wurden [2]. Differenzialdiagnostisch in Betracht gezogen werden sollte eine bakterielle Pneumonie bei hohem Fieber (über 39 °C) bzw. fokalen „crackles“ [3]. Die meisten Guidelines empfehlen die Durchführung eines Thoraxröntgens daher nur bei nichttypischem klinischen Bild bzw. wenn es sich um einen schweren Krankheitsverlauf handelt [4, 7]. Typische radiologische Veränderungen bei akuter Bronchiolitis sind diskrete peribronchiale bzw. parenchymatöse Infiltrate, Überblähungsareale sowie (vorwiegend in den Oberlappen lokalisierte) **Atelektasen**

Atelektasen

([2]; Abb. 1).

**Figure 1:**
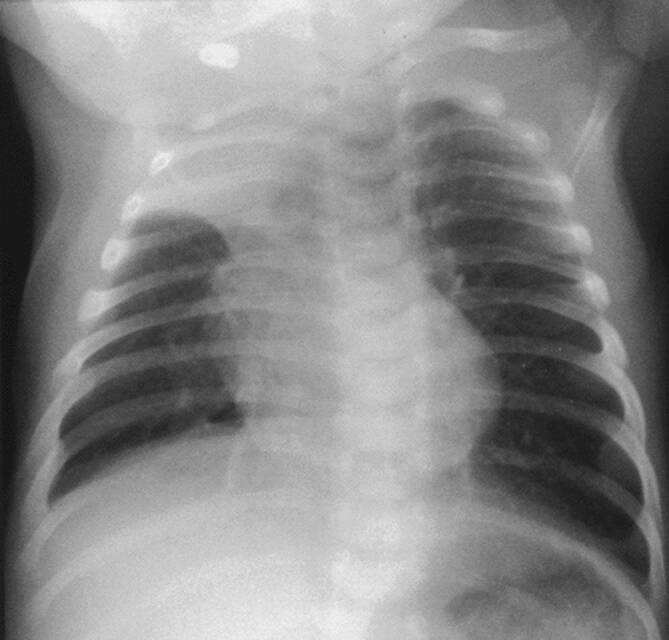

##### Cave

Die radiologischen Veränderungen können einer Pneumonie ähneln und damit zu unnötigen Antibiotikagaben verleiten.

Inwieweit die **Sonographie**

Sonographie

der Lungen zukünftig eine Bedeutung für die Diagnosestellung oder Beurteilung des Schweregrads einer akuten Bronchiolitis haben wird, ist derzeit noch nicht geklärt [7]. Kleinere Studien zeigten eine gute Korrelation zwischen klinischem Bild und sonographischen Befunden; die Spezifität der Sonographie ist möglicherweise höher als die des Thoraxröntgens [7].

#### Laboruntersuchungen

Signifikante bakterielle Sekundärinfektionen sind bei Kindern mit akuter Bronchiolitis äußerst selten [7]. Blutbilduntersuchungen und Blutkulturen werden in Guidelines daher nicht routinemäßig empfohlen [1]. Nur bei sehr jungen Säuglingen wird im Rahmen einer **Fieberabklärung**

Fieberabklärung

ein Blutbild empfohlen; Blutkulturen sollen nur bei **Sepsisverdacht**

Sepsisverdacht

angelegt werden [7]. **Blutgasanalysen**

Blutgasanalysen

und die Bestimmung der Serumelektrolyte können hilfreich sein, um eine respiratorische und/oder metabolische Entgleisung quantifizieren zu können; sie sind daher in schweren Fällen indiziert, nicht aber routinemäßig durchzuführen [2].

Das gleichzeitige Auftreten einer Harnwegsinfektion mit einer akuten Bronchiolitis wird mit 1–7 % angegeben [7]. Daher wird beispielsweise in den Guidelines des Paediatric Research in Emergency Departments International Collaborative (PREDICT) Network empfohlen, bei Säuglingen im Alter unter 2 Monaten mit Fieber über 38 °C und klinischer Unsicherheit eine **Harnanalyse**

Harnanalyse

zu erwägen [8]. Eine rezente Metaanalyse, bei der eine positive Harnanalyse (Nitrit-positiv oder Pyurie) in die Definition einer Harnwegsinfektion inkludiert wurde, ergab eine geschätzte Prävalenz einer Harnwegsinfektion gleichzeitig mit einer akuten Bronchiolitis von 0,8 %, was unter dem Schwellenwert für die Notwendigkeit einer routinemäßigen Testung läge [9]. Harnuntersuchungen bei Kindern mit akuter Bronchiolitis werden daher in Leitlinien routinemäßig nicht empfohlen [10].

#### Virusnachweis

Die meisten Richtlinien empfehlen keine routinemäßige Virustestung, da das Ergebnis bislang weder eine Vorhersagekraft über das Outcome der Erkrankung noch eine therapeutische Konsequenz hatte [7, 10]. Es konnte jedoch beobachtet werden, dass die Krankheitsdauer kürzer ist, wenn die akute Bronchiolitis durch andere Viren als RSV verursacht ist [4]. Studien deuten auch darauf hin, dass eine **hohe Viruslast**

hohe Viruslast

bei RSV-Infektion mit einem schwereren Krankheitsverlauf assoziiert sein könnte [7]. Ein unterschiedliches Ansprechen auf medizinische Interventionen abhängig vom auslösenden Virus konnte bisher nicht eindeutig nachgewiesen werden [6]. Manche Richtlinien empfehlen eine Testung auf RSV, um Patienten entsprechend kohortieren zu können und um epidemiologische Fragestellungen zu beantworten[10].

### Risikofaktoren für schwere Krankheitsverläufe

Säuglinge mit einem chronologischen Alter unter 3 Monaten stellen eine Risikogruppe für schwere Verlaufsformen einer akuten Bronchiolitis dar [4]. Die höchsten Hospitalisierungsraten bei RSV-Bronchiolitiden treten im Alter zwischen einem und 3 Monaten auf [6]. In einer prospektiven populationsbasierten amerikanischen Studie konnte gezeigt werden, dass **Frühgeborene**

Frühgeborene

mit einem Gestationsalter unter 30 Schwangerschaftswochen (SSW) ein deutlich höheres Risiko für eine Hospitalisierung haben als Termingeborene [11]. Gründe hierfür könnten eine verminderte transplazentare Immunglobulin-G-Übertragung bei extremer Frühgeburtlichkeit [6] oder eine veränderte Pathophysiologie der Erkrankung sein [1]. Als weitere Gründe für schwerere Erkrankungsverläufe werden Immundefizienzen, neurologische Erkrankungen, angeborene Anomalien sowie chronische Herz- und Lungenerkrankungen (einschließlich bronchopulmonale Dysplasie, BPD) mit Beeinträchtigung der respiratorischen Kapazität gesehen [3, 4, 8, 10]. **Tabakrauchexposition**

Tabakrauchexposition

ist mit einer höheren Hospitalisierungsrate und schwereren Krankheitsverläufen bei RSV-induzierter akuter Bronchiolitis assoziiert [3, 7]. In den australischen Richtlinien wird als weiterer Risikofaktor eine **Stilldauer**

Stilldauer

unter 2 Monaten angeführt, und in einigen anderen nationalen Richtlinien werden auch Luftverschmutzung und Armut als mögliche Ursachen für schwerere Krankheitsverläufe erwähnt [10]. **Kleinere Atemwege**

Kleine Atemwege

im frühen Säuglingsalter (durch Frühgeburtlichkeit, männliches Geschlecht und/oder intrauterine Tabakexposition) resultieren in einem erhöhten Risiko für schwere, prolongierte Verläufe [2]. Inkonsistente Resultate ergaben Studien bezüglich der Frage, ob Kinder mit zystischer Fibrose oder Down-Syndrom (unabhängig von Herzfehlern) eine erhöhte Hospitalisierungsrate bei RSV-Infektion haben [6].

### Kriterien für eine Hospitalisierung

Die überwiegende Zahl der akuten Bronchiolitiden zeigt einen milden klinischen Verlauf, sodass nur bei 2–3 % der erkrankten Säuglinge eine Hospitalisierung erforderlich ist [2]. Die Notwendigkeit einer Hospitalisierung hängt in erster Linie vom **Erkrankungsschweregrad**

Erkrankungsschweregrad

ab [2]. Zusätzlich sind geografische Faktoren (beispielsweise ein langer Anfahrtsweg zum Krankenhaus) und **soziale Aspekte**

soziale Aspekte

(Adäquate Versorgung gewährleistet? Kann eine Verschlechterung des Zustands rechtzeitig erkannt werden?) zu beachten [3]. Risikofaktoren für schwere Krankheitsverläufe sind auch bei primär milder Symptomatik zu berücksichtigen [8]. Nach den Leitlinien des National Institute for Health and Care Excellence (NICE) sollen Kinder jedenfalls hospitalisiert werden, wenn eines der folgenden Kriterien zutrifft: Apnoe (berichtet oder beobachtet), unzureichende Flüssigkeitszufuhr (entsprechend 50–75 % der normalen Menge), persistierend schwere Atemnotsymptomatik (mit deutlichen Einziehungen bzw. Atemfrequenzen über 70/min) oder persistierend eine Sauerstoffsättigung unter 92 % bei Raumluft [3]. Aktuellere Evidenz deutet allerdings darauf hin, dass sonst stabile Kinder mit akuter Bronchiolitis möglicherweise ebenfalls intermittierend Hypoxämien aufweisen [7]. Damit ist zu hinterfragen, ob überhaupt, und wenn ja, welche **Sauerstoffsättigungsgrenze**

Sauerstoffsättigungsgrenze

als alleiniges Kriterium für eine Hospitalisierung angewendet werden soll [7]. Aufgrund des variablen klinischen Bilds werden Kinder oft stationär aufgenommen, auch wenn sie keine schwerwiegenden Symptome aufweisen, da nicht unbedingt vorhersehbar ist, ob im weiteren Verlauf unterstützende Maßnahmen notwendig werden [6]. Für die Entscheidung über eine Hospitalisierung ist das **Krankheitsstadium**

Krankheitsstadium

zu berücksichtigen; gerade zu Beginn der Erkrankung soll evtl. eine erneute medizinische Begutachtung veranlasst werden, wenn ein Kind nicht stationär aufgenommen wird [8].

#### Merke.

Stadium und Schweregrad der Erkrankung, soziale Aspekte sowie Risikofaktoren für schwere Krankheitsverläufe sollten bei der Entscheidung über eine Hospitalisierung berücksichtigt werden.

### Therapie

Die wichtigsten Therapieansätze sind rein **supportive Maßnahmen**

supportive Maßnahmen

und bestehen aus minimalem Handling sowie Sicherung einer ausreichenden Hydratation und Oxygenierung (durch Sauerstoffgabe und, wenn notwendig, Atemunterstützung, [2]). Der Krankheitsverlauf kann durch keine der verfügbaren Therapien verkürzt werden [6]. Intensivmedizinische Behandlungsoptionen werden in diesem Beitrag nicht thematisiert.

#### Flüssigkeitszufuhr

Bei unzureichender peroraler Hydratation soll die Flüssigkeitszufuhr i.v. oder mittels **transnasaler Magensonde**

transnasaler Magensonde

erfolgen [4, 8]. Die NICE-Leitlinien erwähnen neben der transnasalen Route auch die Möglichkeit einer **oralen Magensonde**

oralen Magensonde

und empfehlen, eine Flüssigkeitssubstitution via Sonde gegenüber einer i.v.-Flüssigkeitssubstitution zu präferieren [3]. Bei Kindern mit schwerer akuter Bronchiolitis besteht ein erhöhtes Risiko für eine inadäquate Sekretion von antidiuretischem Hormon (**SIADH**

SIADH

oder **Schwartz-Bartter-Syndrom**

Schwartz-Bartter-Syndrom)

und konsekutive Ödembildung [2]. Bei i.v.-Verabreichung von hypotonischen Lösungen steigt das Risiko einer **iatrogenen Hyponatriämie**

iatrogenen Hyponatriämie;

daher scheinen isotonische Lösungen zur Flüssigkeitssubstitution sicherer [4].

#### Nasales Absaugen

Es existiert keine randomisierte kontrollierte Studie zu nasalem Absaugen bei akuter Bronchiolitis [7]. Da Säuglinge **obligate Nasenatmer**

obligate Nasenatmer

sind, könnte die Verminderung von Sekret in der Nase zu einer Verbesserung der Atmung beitragen. Andererseits verursacht nasales Absaugen **Stress**

Stress,

der im Sinne des minimalen Handling vermieden werden soll [3]. Die Empfehlungen zu nasalem Absaugen sind in nationalen Guidelines sehr unterschiedlich; wenn überhaupt, wird zumeist nur **oberflächliches Absaugen**

oberflächliches Absaugen

empfohlen [7].

#### Sauerstoffsupplementierung

In einer doppelblinden, randomisierten Studie wurden bei hospitalisierten Kindern mit akuter Bronchiolitis Zielgrenzen der Sauerstoffsättigung von 94 % und 90 % verglichen. Es konnte gezeigt werden, dass eine Sauerstoffsupplementierung bei einer Sauerstoffsättigung unter 90 % genauso sicher und effektiv ist wie eine Supplementierung bereits bei einer Sauerstoffsättigung unter 94 %. Weitere Resultate waren eine **kürzere Hospitalisierungsdauer**

kürzere Hospitalisierungsdauer

und keine Unterschiede bei den Nebenwirkungen [12]. Die Leitlinie der American Academy of Pediatrics (AAP) empfiehlt 90 % als Sauerstoffsättigungsgrenze [4]. Britische und australische Leitlinien empfehlen eine Sauerstoffsupplementierung bei unter 92 %iger Sauerstoffsättigung. In der australischen Leitlinie wird als Begründung angegeben, dass es keine Langzeitstudien bezüglich des entwicklungsneurologischen Outcome bei Säuglingen mit akuter Bronchiolitis gibt und damit **keine Langzeitevidenz**

keine Langzeitevidenz

für die Sicherheit von Sauerstoffsättigungsgrenzen unter 92 % vorhanden ist [3, 8].

#### Nasale „High-flow“-Therapie

In den letzten Jahren wurden zahlreiche Studien zur Anwendung von „high flow nasal cannula“ (HFNC) bei akuter Bronchiolitis publiziert. Es konnte gezeigt werden, dass durch diese Maßnahme die Notwendigkeit von **Intubationen**

Intubation

reduziert werden kann [7] Darüber hinaus gibt es Evidenz dafür, dass HFNC sicher und effektiv auch auf **Normalstationen**

Normalstation

angewendet werden kann [13]. Laut einem aktuellen Review, der nur randomisierte kontrollierte Studien analysiert hat, ist HFNC zwischen herkömmlicher Sauerstoffsupplementation und nasalem „continuous positive airway pressure“ (CPAP) zu positionieren und scheint nicht mit einer Verringerung der stationären Aufenthaltsdauer einherzugehen [14].

#### Physiotherapie

Ein systematischer Cochrane-Review aus dem Jahr 2016 kam zum Schluss, dass die Anwendung physiotherapeutischer Techniken bei akuter Bronchiolitis keine Verbesserung in Bezug auf die Schwere der Erkrankung, Hospitalisierungs‑/Krankheitsdauer oder Sauerstoffbedarf bringt und demzufolge nicht routinemäßig erfolgen soll. Nur die Technik einer **langsamen assistierten Exspiration**

langsamen assistierten Exspiration

konnte eine sofortige, vorübergehende Besserung bei mittelschwer Erkrankten bewirken, jedoch ohne Einfluss auf die Krankheitsdauer [15].

#### Hypertonische Kochsalzlösung

Anfänglich wurden in einigen Studien eine verkürzte Hospitalisierungsdauer (in einem Cochrane-Review aus dem Jahr 2013 um einen Tag [4]) und vorübergehende klinische Verbesserung durch die **Inhalation**

Inhalation

von hypertonischer Kochsalzlösung bei akuter Bronchiolitis berichtet [7]. In aktuellerer Literatur sind die Resultate aber widersprüchlich [7]. In einem aktualisierten Cochrane-Review aus dem Jahr 2017 wurde nur mehr eine Verkürzung der stationären Aufenthaltsdauer um 10 h durch die Inhalation von hypertonischer im Vergleich zu isotonischer Kochsalzlösung festgestellt. Weitere Resultate dieser Analyse waren, dass die Anwendung von hypertonischer Kochsalzlösung keine schwerwiegenden Nebenwirkungen hat, und wenn eine Inhalation präklinisch bzw. in der Notaufnahme erfolgt, die Hospitalisierungsrate um 14 % reduziert werden kann. Die Autoren kommen aber aufgrund der schwachen bis moderaten Evidenz zum Schluss, dass weitere Studien notwendig sind, um die Vorteile einer Anwendung von hypertonischer Kochsalzlösung bei Bronchiolitis zu bestätigen [16]. In vielen Studien wurde hypertonische mit isotonischer Kochsalzlösung verglichen. Ob die Gabe von **isotonischer Kochsalzlösung**

isotonischer Kochsalzlösung

als Placebo geeignet ist oder per se bereits eine Therapie darstellt, hinterfragt ein aktueller systematischer Review, der zeigen konnte, dass die Inhalation von isotonischer Kochsalzlösung möglicherweise zu einer kurzzeitigen klinischen Verbesserung führt [17]. Die Empfehlungen in Leitlinien variieren. Laut den amerikanischen Leitlinien (allerdings aus dem Jahr 2014) kann die Anwendung von hypertonischer Kochsalzlösung bei hospitalisierten Kindern in Betracht gezogen werden, soll aber in der Notaufnahme nicht erfolgen [4]. Die britischen und noch aktuelleren australischen Leitlinien empfehlen eine Therapie mit hypertonischer Kochsalzlösung bei akuter Bronchiolitis nicht [3, 8].

#### Bronchodilatatoren

Die Anwendung von Bronchodilatatoren wie **Salbutamol**

Salbutamol

(Albuterol) führt zu keiner Verkürzung der Krankheitsdauer, reduziert weder die Rate noch die Dauer einer Hospitalisierung und führt auch nicht zu einer Verbesserung der Sauerstoffsättigung, wie ein systematischer Cochrane-Review aus dem Jahr 2014 zeigen konnte [18]; diese Ergebnisse sind aufgrund der Pathophysiologie der akuten Bronchiolitis nicht verwunderlich. Angesichts möglicher **Nebenwirkungen**

Nebenwirkung

(Tachykardie, Sauerstoffsättigungsabfälle und Tremor) und entstehender Kosten sind Bronchodilatatoren daher nicht effizient in der routinemäßigen Therapie der akuten Bronchiolitis [18]. Die Anwendung von Bronchodilatatoren in der Therapie der akuten Bronchiolitis wird daher nicht empfohlen [3, 4, 8]. Große, randomisierte, multizentrische Studien zeigten keine Verbesserung des Outcome bei ambulant betreuten oder hospitalisierten Kindern mit akuter Bronchiolitis nach Anwendung von **Epinephrin**

Epinephrin

[6]. Die meisten Guidelines empfehlen daher überhaupt keine oder zumindest keine routinemäßige Anwendung von vernebeltem Epinephrin [7].

#### Kortikosteroide

Ein systematischer Cochrane-Review aus dem Jahr 2014 und große, randomisierte, multizentrische Studien erbrachten eine **klare Evidenz**

klare Evidenz

dafür, dass die Anwendung von Kortikosteroiden bei akuter Bronchiolitis keinen Vorteil bringt [4]. Im Speziellen gibt es auch keine Evidenz dafür, dass Kinder mit atopischen Manifestationen oder Atopie in der Familienanamnese von einer Kortikosteroidtherapie bei akuter Bronchiolitis profitieren [7].

#### Leukotrienrezeptorantagonisten

Im Atemwegssekret von Patienten mit akuter Bronchiolitis wurden **erhöhte Leukotrienwerte**

erhöhte Leukotrienwerte

nachgewiesen [2]. Aufgrund der vorliegenden Evidenz ist laut einem systematischen Cochrane-Review aus dem Jahr 2015 jedoch keine definitive Schlussfolgerung bezüglich der Wirkung von Leukotrienrezeptorantagonisten auf Hospitalisierungsdauer oder Schweregrad bei Säuglingen mit akuter Bronchiolitis möglich [19].

#### Antibiotika

Antibiotika werden bei jungen Säuglingen mit akuter Bronchiolitis aus Angst vor unentdeckten bakteriellen Infektionen zu häufig verordnet [4]. Sekundäre bakterielle Infektionen bei akuter Bronchiolitis sind sehr selten [8]. Das Risiko für eine Sepsis oder Meningitis bei akuter Bronchiolitis beträgt unter 1 % [7]. Antibiotika sollten daher nur bei einer gesicherten bakteriellen Infektion oder bei starkem Verdacht auf eine bakterielle Infektion eingesetzt werden [4]. Gerechtfertigt kann eine antibiotische Therapie sein, wenn Kinder mit **Atemversagen**

Atemversagen

intubiert und mechanisch beatmet werden [4]. Ob Antibiotika persistierende Atemwegssymptome nach akuter Bronchiolitis (in den ersten 6 Monaten nach Erkrankung) reduzieren können, wurde in einem systematischen Cochrane-Review aus dem Jahr 2017 untersucht. Nach Meinung der Autoren gibt es keine ausreichende Evidenz für eine Therapie mit Antibiotika bezüglich dieser Fragestellung [15].

##### Merke.

In der Therapie der akuten Bronchiolitis wird die routinemässige Anwendung von Bronchodilatatoren, Kortikosteroiden, Leukotrienrezeptorantagonisten oder Antibiotika nicht empfohlen.

### Prävention

Zur Prävention einer RSV-Infektion ist **Palivizumab**

Palivizumab,

ein humanisierter monoklonaler IgG_1_-Antikörper zugelassen [6]. Aufgrund der hohen Kosten dieser **passiven Immunisierung**

passiven Immunisierung

(pro Saison sind bis zu 5 Dosen i.m. zu applizieren) soll eine Anwendung nur bei Hochrisikokindern erfolgen [2]. Die Empfehlungen zu den Indikationen für Palivizumab variieren [20]. Laut den nordamerikanischen Leitlinien der AAP soll eine RSV-Prophylaxe im 1. Lebensjahr bei allen Frühgeborenen mit einem Gestationsalter unter 29 Schwangerschaftswochen (SSW), bei Frühgeborenen unter 32 SSW mit BPD und bei Kindern mit hämodynamisch wirksamen Herzfehlern durchgeführt werden [4]. Dies empfehlen auch die Arbeitsgemeinschaft der Wissenschaftlichen Medizinischen Fachgesellschaften e. V. (AMWF) und die Österreichische Gesellschaft für Kinder- und Jugendheilkunde (ÖGKJ). Darüber hinaus inkludieren sowohl die deutschen als auch österreichischen Empfehlungen in der Risikobewertung für eine RSV-Infektion Faktoren wie Kinderkrippenbesuch, ältere Geschwister, schwere neurologische Erkrankungen, Entlassung von einer Neonatologie kurz vor oder während der RSV-Saison und chronologisches Alter zu Beginn der RSV-Saison (der Risiko-Score der ÖGKJ enthält darüber hinaus die Faktoren Mehrling, niedriges Gewicht, Tabakbelastung und Sozialstatus). Je nach resultierender **individueller Risikoabschätzung**

individueller Risikoabschätzung

wird auch für Frühgeborene mit einem Gestationsalter von 29 + 0 bis 34 + 6 SSW eine RSV-Prophylaxe empfohlen [20]. Des Weiteren wird bei therapiepflichtiger BPD im 2. Lebensjahr laut ÖGKJ und AAP, bzw. bis 3 Monate vor der RSV-Saison laut AMWF, eine Prophylaxe für die 2. RSV-Saison empfohlen.

## Obstruktive Bronchitis

### Definition, Ätiologie und Epidemiologie

Der Begriff obstruktive Bronchitis bezeichnet eine in der Regel virale Infektion der unteren Atemwege mit Atemwegsobstruktion (in erster Linie der mittleren und größeren Bronchien) und dem Leitsymptom **„wheezing“**

„wheezing“.

Weitere typische Symptome und Zeichen sind Husten, Tachypnoe und juguläre bzw. thorakale Einziehungen. Bei schwereren Verläufen kommt es zu Sauerstoffsättigungsabfällen; bei massiver Überblähung der Lungen kann möglicherweise auch kein exspiratorisches Giemen bzw. Pfeifen auskultierbar sein. Knisterrasseln wie bei einer akuten Bronchiolitis (mit Affektion v. a. der kleinen Bronchien und Bronchiolen) ist typischerweise nicht vorhanden. In manchen Fällen kann eine klare Abgrenzung der obstruktiven Bronchitis zu einer Bronchiolitis schwierig sein, denn Überschneidungen sind möglich. Wie eingangs erwähnt, wird in vielen Studien keine Differenzierung zwischen den beiden Krankheitsbildern bei Patienten in den ersten 2 Lebensjahren durchgeführt, sondern beide werden als akute Bronchiolitis bezeichnet. Ebenfalls nicht immer einfach ist bei Kleinkindern eine Unterscheidung zwischen episodischen, viral induzierten obstruktiven Bronchitiden und einem Asthma bronchiale [2].

Ursache einer obstruktiven Bronchitis sind in der Regel Infektionen durch Viren, v. a. Rhinoviren (häufigste Erreger im 2. Lebensjahr [5]) und RSV, außerdem humane Metapneumoviren, Coronaviren, Parainfluenzaviren, Adenoviren und Influenzaviren.

Etwa jedes 3. Kind hat zumindest eine obstruktive Bronchitis vor dem vollendeten 3. Lebensjahr [21, 22]. Obstruktive Bronchitiden sind ab dem 6. Lebensjahr selten, viel wahrscheinlicher sind im Schulalter infektionsassoziierte Exazerbationen bei **Asthma bronchiale**

Asthma bronchiale,

an dem etwa 10 % aller Kinder erkrankt sind [22].

### Klinischer Verlauf, Diagnose und Differenzialdiagnosen

Nach Schnupfen, trockenem Husten und evtl. geringgradigem Fieber treten Zeichen erhöhter Atemarbeit, bei schwereren Verläufen auch Sauerstoffsättigungsabfälle und Atemnot auf. Ein exspiratorisch pfeifendes Atemgeräusch ist charakteristisch für die obstruktive Bronchitis. Normalerweise sistiert die Symptomatik nach einer bis 2 Wochen. Vollkommen asymptomatische Intervalle zwischen Infektionen sind von diagnostischer Bedeutung für die Abgrenzung zu Asthma bronchiale [2].

Die Diagnose obstruktive Bronchitis wird in erster Linie durch Anamnese und klinische Untersuchung gestellt. Wie bei der akuten Bronchiolitis wird auch bei einer obstruktiven Bronchitis nicht empfohlen, routinemäßig Laboruntersuchungen, ein Thoraxröntgen oder einen Virusnachweis durchzuführen. Bei schweren Verläufen (mit beispielsweise Verdacht auf Atelektase) oder zur differenzialdiagnostischen Abklärung kann eine Röntgenuntersuchung im Einzelfall erwogen werden [2]. Der Nachweis von **Sensibilisierungen**

Sensibilisierungen

gegenüber Nahrungsmittel- bzw. Inhalationsallergenen kann hilfreich sein, um eine atopische Diathese bzw. bei entsprechendem klinischen Bild eine Allergie festzustellen [22]. Ob die Bestimmung der Eosinophilenzahl im peripheren Blut von diagnostischem und evtl. therapeutischem Nutzen ist, muss noch in weiteren Studien geklärt werden. Eine **Bluteosinophilie**

Bluteosinophilie

wird als Hinweis auf atopische Manifestationen gesehen und kann mit einer eosinophilen Entzündung der unteren Atemwege assoziiert sein [21].

Das Leitsymptom der obstruktiven Bronchitis (**„wheezing“**

„wheezing“)

kann auch eine Reihe anderer zugrunde liegender Ursachen haben. Daher muss bei atypischem klinischen Bild an entsprechende Differenzialdiagnosen (Tab. 2) gedacht werden.

**Table 2:** 

Klinische Warnzeichen	Differenzialdiagnosen
Symptome ab Geburt	Tracheobronchomalazie, primäre ziliäre Dyskinesie (PCD), zystische Fibrose (CF), „chronic lung disease of infancy“ (CLD), gastroösophagealer Reflux
Feuchter produktiver Husten	CF, PCD, Immundefizienz, Pneumonie, Tuberkulose
Plötzliches Auftreten ohne vorbestehende Probleme	Fremdkörperaspiration
Stridor	Laryngitis, Tracheitis, Laryngo- oder Tracheomalazie
Vorwiegend nächtliche Beschwerden	Gastroösophagealer Reflux, Problematik der oberen Atemwege („postnasal drip“)
Gedeihstörung	CF, Immundefizienz
Keine asymptomatischen Intervalle	Tracheobronchomalazie, CLD, Malformationen (z. B. Gefäßring)

### Risikofaktoren für schwere Verlaufsformen und häufige obstruktive Bronchitiden

Störungen der Atemwegsentwicklung (wie beispielsweise bei Frühgeburtlichkeit, insbesondere mit BPD, bei kongenitalen Atemwegsanomalien oder intrauteriner Exposition gegenüber Tabakrauch) sind prädisponierende Faktoren für schwerere Verläufe von obstruktiven Bronchitiden oder deren vermehrtes Auftreten. Adipositas und eine **überproportionale Gewichtszunahme**

überproportionale Gewichtszunahme

im Säuglingsalter können durch einen negativen Einfluss auf die Atemwegsmechanik auch zu einer Aggravation von „Wheezing“-Episoden beitragen. Durch das Lungenwachstum verlieren diese, die Atemwegsentwicklung und -mechanik betreffenden Faktoren zunehmend an Bedeutung. Im Unterschied dazu sind Immundysfunktionen und Störungen der **epithelialen Funktion**

epithelialen Funktion

(v. a. Atopie, aber auch beispielsweise Immunmangelsyndrome) bis in das Schulalter präsente Risikofaktoren. Gegenüber **Luftschadstoffen**

Luftschadstoffen

bzw. Tabakrauch exponierte und nichtgestillte Kinder haben ebenfalls eine höhere Wahrscheinlichkeit für schwerere Verlaufsformen und das häufigere Auftreten von obstruktiven Bronchitiden [2].

### Klassifikation von „Wheezing“-Episoden

In der Tucson Children’s Respiratory Study, einer longitudinalen Studie an einer nordamerikanischen Geburtskohorte, konnten 4 Gruppen von Kindern unterschieden werden: (1) Kinder, die nie eine obstruktive Atemwegserkrankung mit „wheezing“ aufwiesen; (2) Kinder mit transientem „wheezing“ in den ersten 3 Lebensjahren; (3) Kinder, bei denen „wheezing“ erstmalig nach dem 3. Lebensjahr auftrat; und (4) Kinder, die bereits in den ersten 3 Lebensjahren und auch im 4. bis 6. Lebensjahr Symptome zeigten [23]. Diese Einteilung ist zwar von epidemiologischem Interesse, jedoch für das klinische Management individueller Patienten ungeeignet. In weiteren Kohortenstudien konnte eine Assoziation zwischen persistierendem „wheezing“ und **Atopie**

Atopie

bzw. mütterlichem Asthma und Eosinophilie im Serum festgestellt werden. Eine mögliche Klassifikation unterscheidet daher zwischen Kindern mit und ohne Atopie. Im Jahr 2008 prägte eine Task Force der European Respiratory Society (ERS) die Begriffe „episodic viral wheeze“ (rein virusassoziierte obstruktive Atemwegserkrankungen) im Gegensatz zu „multiple trigger wheeze“ („wheezing“ wird nicht nur durch Virusinfektionen ausgelöst und Symptome sind auch in infektionsfreien Intervallen vorhanden, [24]). Diese Klassifikation nach **Trigger-Faktoren**

Trigger-Faktoren

wird immer noch häufig verwendet; allerdings gibt es mittlerweile klare Evidenz dafür, dass die Phänotypen im zeitlichen Verlauf nicht stabil sind [25, 26]. Des Weiteren wurden in dieser Klassifikation weder der Schweregrad noch die Häufigkeit von obstruktiven Bronchitiden berücksichtigt, die aber wesentliche Faktoren für Therapieentscheidungen sind.

### Therapie

Die medikamentöse inhalative Therapie im Kleinkindalter soll mithilfe eines Dosieraerosols und einer Vorschaltkammer durchgeführt werden. Nur wenn dies nicht möglich ist, kann eine **inhalative Therapie**

inhalative Therapie

auch mit einem Kompressionsvernebler durchgeführt werden. Regelmäßige Schulungen zur korrekten Anwendung der inhalativen Therapie sowie Therapieadhärenz sind wesentliche Erfolgsfaktoren [21].

Obwohl in einem Cochrane Review aus dem Jahr 2009 keine klare Evidenz für einen Effekt von **kurz wirksamen β**_**2**_**-Sympathikomimetika**

β_2_-Sympathikomimetika

bei Kindern unter 2 Jahren nachgewiesen werden konnte, sind **kurz wirksame β**_**2**_**-Agonisten**

kurz wirksame β_2_-Agonisten

die 1. therapeutische Wahl bei einer akuten obstruktiven Bronchitis. Aufgrund des heterogenen Ansprechens sollte der **individuelle Therapieerfolg**

individuelle Therapieerfolg

jedoch evaluiert werden [26]. Allein angewendet sind **Anticholinergika**

Anticholinergika

weniger effektiv als β_2_-Sympathikomimetika, in Kombination können diese beiden Substanzgruppen einen synergistischen Effekt haben [26]. Lang wirksame β_2_-Mimetika sind für das Säuglings- und Kleinkindalter nicht zugelassen.

Orale Kortikosteroide sollen bei obstruktiven Bronchitiden nicht routinemäßig angewendet werden. Bei hospitalisierten Kindern mit schweren Verläufen kann eine systemische Gabe von Kortikosteroiden erwogen werden (insbesondere wenn bereits der Verdacht auf ein Asthma bronchiale besteht, [21]). Für den Nutzen von längerfristig inhalierten Kortikosteroiden bei rein infektionsassoziierten obstruktiven Bronchitiden gibt es wenig Evidenz. Trotzdem kann bei häufigen und schweren obstruktiven Bronchitiden unabhängig vom Phänotyp zur Symptomkontrolle eine längerfristige medikamentöse Therapie mit **inhalativen Kortikosteroiden (ICS)**

inhalativen Kortikosteroiden (ICS)

versucht werden [21]. Eine Reevaluation einer solchen Therapie ist notwendig [21].

Antibiotika sollen bei obstruktiver Bronchitis nicht routinemäßig, sondern nur in begründeten Ausnahmefällen angewendet werden. Während Studien gezeigt haben, dass bei Kindern mit „Wheezing“-Episoden oft auch bakterielle Erreger nachweisbar sind, ist derzeit noch unklar, ob, und wenn ja, inwieweit diese Pathogene eine kausale Rolle spielen [21].

### Zusammenhang zwischen Bronchiolitis, episodischen viralen obstruktiven Bronchitiden und Asthma

Eine umfassende Diskussion der Literatur zu diesem Thema würde den Rahmen dieses Artikels sprengen. Im Folgenden werden ein paar wesentliche Aspekte festgehalten:

- Das Risiko, an Asthma zu erkranken, ist bei Kindern, die als junge Säuglinge eine schwere akute Bronchiolitis durchgemacht haben (insbesondere wenn durch RSV oder Rhinoviren verursacht) erhöht [6].
- Multiple Sensibilisierungen gegenüber Nahrungsmittel- bzw. Inhalationsallergenen in einem frühen Alter gehen mit einem erhöhten Risiko für schwere obstruktive Atemwegserkrankungen mit Hospitalisierung und Asthma bronchiale einher [26].
- Bei der Entstehung von Asthma spielt eine komplexe Interaktion zwischen Umweltfaktoren und genetischer Disposition eine große Rolle. Identische genetische Faktoren können sowohl mit einer schweren akuten Bronchiolitis als auch mit Asthma bronchiale assoziiert sein [6].
- Die Exposition gegenüber Tabakrauch und anderen Luftschadstoffen ist ein Risikofaktor für eine akute Bronchiolitis, rekurrierende obstruktive Bronchitiden und Asthma bronchiale [10, 21].
- Keine pharmakologische Therapie (einschließlich inhalative Kortikosteroide) im Kleinkindalter kann die Entstehung eines Asthma bronchiale verhindern oder hat einen Einfluss auf den Langzeitverlauf eines Asthmas [21]

## Fazit für die Praxis

- Der typische Auskultationsbefund bei akuter Bronchiolitis ist endinspiratorisches Knisterrasseln; für eine obstruktive Bronchitis ist exspiratorisches Pfeifen charakteristisch. Überschneidungen der beiden Krankheitsbilder sind möglich.
- Die Diagnosen akute Bronchiolitis und obstruktive Bronchitis können durch Anamnese und klinische Untersuchung gestellt werden; Thoraxröntgen und Laboruntersuchungen sind in der Regel nicht erforderlich.
- Da das klinische Bild einer akuten Bronchiolitis besonders am Krankheitsbeginn sehr variabel sein kann, sollten zur Einschätzung des Schweregrads der Erkrankung klinische Befunde mehrfach über einen Beobachtungszeitraum erhoben werden.
- Die Therapie bei akuter Bronchiolitis besteht in supportiven Maßnahmen wie Sicherung von ausreichender Oxygenierung und Hydratation bei minimalem Handling. Routinemäßig nicht empfohlen werden pharmakologische Therapieoptionen.
- Kurz wirksame β_2_-Agonisten sind Therapie der Wahl bei akuter obstruktiver Bronchitis. Eine Inhalationstherapie soll im Säuglings- und Kleinkindalter primär mit Dosieraerosol und Vorschaltkammer erfolgen. Regelmäßige Schulungen zur korrekten Anwendung der Inhalationstherapie sowie eine Evaluierung des individuellen Therapieerfolgs sind erforderlich.

## CME-Fragebogen

Welches der folgenden Symptome und Zeichen ist charakteristisch für eine akute virale Bronchiolitis?

Inspiratorischer Stridor

Inspiratorisches Pfeifen

Inspiratorisches Knisterrasseln

Exspiratorischer Stridor

Exspiratorisches Giemen

Ein 18 Monate altes Kleinkind präsentiert sich mit einer Atemfrequenz von 52/min, interkostalen Einziehungen, einer pulsoxymetrischen Sauerstoffsättigung von 92 % unter Raumluft und einem exspiratorisch giemenden Atemgeräusch beidseits. Anamnestisch bestanden in den Tagen zuvor Schnupfen und subfebrile Temperaturen. Wie lautet die wahrscheinlichste Diagnose?

Asthma bronchiale

Bronchiolitis

Fremdkörperaspiration

Obstruktive Bronchitis

Pneumonie

Welches diagnostische Vorgehen ist geeignet, um eine obstruktive Bronchitis zu diagnostizieren?

Bestimmung von Blutbild und C‑reaktivem Protein

Blutgasanalyse

Messung der Atemfrequenz und Auskultation

Respiratory-syncytial-virus(RSV)-Schnelltest

Thoraxröntgen

Welche Aussage zu inhalativen Bronchodilatatoren in der Therapie der akuten Bronchiolitis ist richtig?

Inhalative Bronchodilatatoren führen zu einer Verkürzung der Krankheitsdauer.

Inhalative Bronchodilatatoren führen zu einer Verbesserung der Sauerstoffsättigung.

Inhalative Bronchodilatatoren reduzieren die Rate und Dauer der Hospitalisierung.

Inhalative Bronchodilatatoren sollten routinemäßig eingesetzt werden.

Inhalative Bronchodilatatoren können zu Tachykardie, Sauerstoffsättigungsabfällen und Tremor führen.

Welche der folgenden Maßnahmen zählt zu den empfohlenen therapeutischen Maßnahmen bei mittelschwerer Bronchiolitis?

Atemphysiotherapie

Bronchodilatatoren inhalativ

Kortikosteroide per os

Leukotrienrezeptorantagonisten per os

Sauerstoffsupplementierung

Welche Symptomatik gilt als eines der Hauptkriterien für eine Hospitalisierung bei akuter Bronchiolitis?

Eine Sauerstoffsättigung von 94 % unter Raumluft

Das Auftreten von Apnoen

Eine Atemfrequenz von 60/min

Eine auf 80 % des Üblichen verminderte Trinkleistung

Das Auftreten von Erbrechen

Welches Virus verursacht am häufigsten obstruktive Bronchitiden im 2. Lebensjahr?

Coronavirus

Humanes Metapneumovirus

Parainfluenzavirus

„Respiratory syncytial virus“

Rhinovirus

Ein 14 Monate altes Kleinkind präsentiert sich erstmalig mit Symptomen bzw. Zeichen einer obstruktiven Bronchitis. Was ist die Therapie der Wahl?

Kortikosteroid per os

Kortikosteroid inhalativ

Kurz wirksamer β_2_-Agonist inhalativ

Lang wirksamer β_2_-Agonist inhalativ

Montelukast per os

Ein 5 Monate altes Kleinkind präsentiert sich in der Notfallambulanz mit einer Atemfrequenz von 70/min, Nasenflügeln, thorakalen Einziehungen und einem auskultatorisch sehr leisen Atemgeräusch. Die Sauerstoffsättigung beträgt 88 % unter Raumluft. Wie lautet Ihre Therapieempfehlung?

Sauerstoffsupplementation und Gabe eines systemischen Kortikosteroids

Sauerstoffsupplementation und Hospitalisation

Sauerstoffsupplementation und Inhalation von Epinephrin

Sauerstoffsupplementation und Inhalation von hypertonischer Kochsalzlösung

Sauerstoffsupplementation und Inhalation von Salbutamol

Wie lauten die deutschen und österreichischen Empfehlungen bezüglich einer „Respiratory-syncytial-virus“(RSV)-Prophylaxe mit Palivizumab im 1. Lebensjahr?

Palivizumab ist indiziert bei allen Frühgeborenen mit einem Gestationsalter <37 SSW.

Palivizumab ist in erster Linie bei männlichen Frühgeborenen indiziert.

Palivizumab ist nur für immunsuffiziente Frühgeborene zugelassen.

Palivizumab ist ein Lebendimpfstoff, der einmalig im 1. Lebensjahr verabreicht wird.

Palivizumab ist bei Frühgeborenen <29 SSW bzw. Frühgeborenen <32 SSW mit bronchopulmonaler Dysplasie (BPD) oder hämodynamisch wirksamem Herzfehler indiziert.
